# Evaluation of liver function tests to identify hepatotoxicity among acute lymphoblastic leukemia patients who are receiving chemotherapy induction

**DOI:** 10.1038/s41598-022-17618-w

**Published:** 2022-08-02

**Authors:** Ayal Tsegaye Mekonnen, Temesgen Gebeyehu Wondmeneh

**Affiliations:** 1 Department of Biomedical, College of Medical and Health Science, Samara University, Semera, Ethiopia; 2 Department of Public Health, College of Medical and Health Science, Samara University, Semera, Ethiopia

**Keywords:** Cancer, Oncology

## Abstract

The effect of induction chemotherapy on liver function in patients with acute lymphoblastic leukemia is not well documented in Ethiopia. This study assessed hepatotoxicity in patients with acute lymphoblastic leukemia who were undergoing induction chemotherapy in Ethiopia. A 1-month cohort study was undertaken in forty patients with acute lymphoblastic leukemia, with measurements taken at the baseline, second, and fourth weeks. A Log 10 transformation was done because of the skewed distribution of liver function tests. Descriptive statistics such as mean and proportion were calculated. A mixed model ANOVA and Bonferroni post hoc test were computed. A *p* value < 0.05 was declared to determine statistical significance. Clinically significant hepatotoxicity was observed in 15% of patients. Mild liver injury occurred in 5% of patients. The mean of all liver function tests increased significantly from pre-induction to post-induction. ALT levels were significantly higher in patients who received blood transfusions, but not in those who did not. Regardless of other factors, ALP level in children is significantly higher than in adults, although total bilirubin in adults is higher than in children. A significant proportion of patients had hepatotoxicity. During chemotherapy induction, the mean of all liver function tests rose significantly, but this elevation of serum liver function tests may be transient. Chemotherapy drugs should be given without causing a significant alteration in serum liver function tests. Continuous monitoring of patients should be required.

## Introduction

Acute lymphoblastic leukemia (ALL) is the most frequent childhood cancer, but adults can be affected as well^1–3^, and it is caused by hereditary links and genetic abnormalities^4^. Chemotherapy induction for acute lymphocytic leukemia (ALL) is used to clear immature white blood cells from the blood and bone marrow and achieve full remission or response. The induction phase of treatment lasts a month^5,6^. Chemotherapy is divided into three phases: the induction phase, the consolidation (or intensification) phase, and the maintenance phase. The goal of treatment is to achieve remission, which is usually defined as a reduction of less than 5% of blast cells in the bone marrow^7^. Induction treatment (protocol 1) began with one week of prednisone and intrathecal methotrexate (protocol 1/phase 1) and was followed by a 4-week regimen of daily prednisone, intrathecal methotrexate on days 15 and 29, weekly vincristine and daunorubicin, and three times per week l-asparaginase^8^. Hepatotoxicity is defined as a liver injury caused by medication exposure that result in altered liver function. Hepatocellular liver injury is a prominent initial elevation of the alanine aminotransferase level, cholestatic, as evidenced by an increase in serum alkaline phosphatase levels, or mixed if both enzymes are elevated. ALT levels exceeding three times the upper limit of normal values and total bilirubin concentrations greater than twice the upper limit are defined as clinically significant abnormalities on liver tests^9–12^. Patients who received four or more chemotherapy drugs were at risk of experiencing adverse medication reactions^13^. In the absence of other more likely causes of elevated liver tests, post-chemotherapy liver test results were defined as drug-induced liver injury if ALT was > 3 times the upper limit of normal and total bilirubin (TB) was > 2–3 times the upper limit of normal^14,15^. Chemotherapy hepatotoxicity often occurs unexpectedly or in an idiosyncratic manner, and previous liver disease increases the risk^16^. During the treatment of acute leukemia, serum total bilirubin, ALT, and AST levels rise in response to chemotherapy drugs^17,18^. The anti-leukemic therapy given for the induction of remission did not induce hepatotoxicity except that the serum SGPT was transiently increased during the induction of remission. Elevation in serum enzyme levels is taken as an indicator of liver injury, whereas increases in bilirubin levels, albumin concentration, and prothrombin time are measures of overall liver function^12^. In Singapore^19^ and the United States^20^, 10.7% and 53.9% of patients with acute lymphoblastic leukemia who received L-asparaginase had hepatic transaminase and grade 3/4 transaminitis, respectively. In a study from the Netherlands, in patients treated according to ALL-1, grade 3/4 hyperbilirubinemia and elevated ALT occurred in 10% and 26% of patients, respectively. Hepatotoxicity occurred in 9.76% of patients treated with the Dana Farber Consortium Protocol (DFCP) in Egypt. Adolescents and young adults had favorable outcomes with DFCP but more toxicity^21^. Greater asparaginase activity levels resulted in statistically significant increases in ALT and bilirubin^22^. Genetic variations in genes that are linked to hepatotoxicity and cardiotoxicity could influence the safety of standard induction therapy in pediatric patients with acute lymphoblastic leukemia^23^. Hepatotoxicity was significantly associated with obesity and hypofibrinogenemia, but not to L-asparaginase doses^24^. Adolescents and young adults are highly susceptible to developing asparaginase liver toxicity^25^. L-asparaginase treatment was associated with acute liver toxicity in patients with acute lymphoblastic leukemia in a Sudanese study. Adults receiving L-asparaginase treatment are more likely than children to develop acute liver toxicity^26^.

Chemotherapy induction associated with hepatotoxicity during the induction phase has never been studied in Ethiopia. Clinicians will be able to use these findings to better monitor and manage patients in the future. Thus, the study aimed to determine whether chemotherapy induction is associated with hepatotoxicity in patients with acute lymphoblastic leukemia in Black Lion Hospital, Ethiopia.

## Methods

### Study area and design

The study was conducted at Black Lion Hospital in Addis Ababa, Ethiopia. The Black Lion Specialized Hospital is affiliated with Addis Ababa University. It is the largest tertiary hospital in the country. From July 1 to September 30, 2019, a cohort study was conducted to evaluate chemotherapy-induced hepatotoxicity in patients with acute lymphoblastic leukemia.

### Patient information

Patients over the age of one year who were newly diagnosed with acute lymphoblastic leukemia at Black Lion Hospital between July 1 and September 30, 2019, were included. The same study group is divided into three groups based on the dosages of induction chemotherapy drugs. These were the baseline study group, the second-week induction study group, and the fourth-week induction study group.

#### Eligible criteria

Patients have been admitted for at least one month and have not yet started induction therapy.

#### Variables and operational definition

Dependent variables: Hepatotoxicity.

Independent variables: Age, sex, alcohol intake, weight, blood transfusion and chemotherapy induction drugs.

Induction protocol: All patients were treated in Modified Berlin-Frankfurt-Munster 95 dose schedule in Phase A induction chemotherapy^27^ (Table 1).

**Table 1:** Modified Berlin-Frankfurt-Munster 95 dose schedule in Phase A induction chemotherapy.

Duration	Drugs	Dose	Route of administration	Days
4 weeks	Prednisone	60 mg/m^2^/day	Oral	Day 1–28
	Vincristine	1.5 mg/m^2^/dose	IV	Day 8, 15, 22, 28
	Doxorubicin	30 mg/m^2^/dose	IV	Day 8, 15, 22, 28
	L-asparaginase	5000 IU/m^2^	IM	Day 8, 15, 22, 28

Children are defined as those who are under the age of 18 years^28^.

Hepatotoxicity is defined as any of the following: ALT levels greater than or equal to a fivefold increase above the baseline, or ALT levels greater than or equal to a threefold increase, with a simultaneous increase in bilirubin concentration greater than twofold of the baseline (Table 2)^29^.

**Table 2:** The drug induced liver injury index^29^.

Grading	Severity	Description
1	Mild	Elevated alanine aminotransferase/alkaline phosphatase (ALT/ALP) levels reaching hepatotoxicity criteria*, but bilirubin levels were < 2 × the baseline
2	Moderate	Elevated ALT/ALP levels reaching hepatotoxicity criteria*, as well as bilirubin levels of ≥ 2 × the baseline, or symptomatic hepatitis
3	Sever	Elevated ALT/ALP concentrations reaching hepatotoxicity criteria*, bilirubin concentration ≥ 2 × the baseline, and one of the following: normalized ratio ≥ 1.5, Ascites and/or encephalopathy, disease duration < 26 weeks, absence of underline cirrhosis, and other organ failures due to hepatotoxicity
4	Fatal	Death or transplantation due to hepatotoxicity

*Criteria for hepatotoxicity are defined in the preceding definition.

### Measurements

The same groups' liver function tests were performed three times a month at baseline, the second week after induction, and the fourth week after induction.

#### Sample size and sampling method

Each of the three measurements included 40 patients, totaling 120. Purposive sampling was used to select study participants.

### Data collection and blood sampling procedures

#### Data collection

During the data collection period, all participants who were willing to receive chemotherapy induction were included indiscriminately. Informed consent was obtained from each study participant after a brief explanation of the study aim. Background information was collected by structured interview questionnaires. Then, serum blood samples were taken.

### Blood sample data collection and processing

Blood samples were taken from participants in 5 ml serum separator tubes once before induction and twice after chemotherapy induction in the 2nd and 4th weeks. After keeping the tube for 20 min to clot, the specimen was centrifuged at 1500 rpm for 5 min, and the serum was separated and stored at 40° until an assay was conducted. The liver function tests were conducted on serum at the Ethiopian Public Health Institute.

#### Test analysis

The principle of a spectrophotometer analyzed the liver function test to measure the absorption spectrum of the analyst at every wavelength. A Roche-COBAS Integra® 400 automated chemistry analyzer was used. All tests were performed based on the manufacturer's protocol.

#### Data quality assurance

There was a fair procedure for the selection of study participants and measurement of outcomes.

#### Data analysis

The data were entered into a database sheet in SPSS version 23. The Shapiro–Wilk test showed that the distributions of all liver function tests were skewed. As a result, a log 10 transformation was computed. The mean and standard deviation were calculated using a log 10 transform. The mean and standard deviations (SD) were then back-transformed. A mixed ANOVA model is used to examine the relationship between independent factors and times (baseline, second week of induction, and fourth week of induction). Greenhouse–Geisser was applied when the assumption of sphericity was violated. A Bonferroni post hoc test was conducted when the F-test was significant. If there is an interaction between the independent variables and the dependent variable, a splitting method was used to determine which group the association occurred in. A *p* value < 0.05 was declared to determine whether the difference was statistically significant.

### Ethical consideration

Ethical clearance was obtained from the ethics review board (ERB) of Addis Ababa University's health science college. All methods were performed in accordance with the relevant guidelines and regulations. No one was harmed because of participating in this study. After a brief explanation of the study objective, informed consent was obtained from adults and the child’s guardian. In addition to parental informed consent, the child’s informed assent was obtained as much as the child’s ability. The right of participants to withdraw at any time was respected. By eliminating identifiers from questionnaires, confidentiality was preserved.

## Results

### Socio-demographic characteristics of patients with acute lymphoblastic leukaemia

A total of forty patients with acute lymphoblastic leukemia were included in this study. Out of these, 55% of them were males. The median age of patients was 14 years (range 2–52 years). Most of the participants were children, with 40% of those aged 1–9 years. Over 85% of patients had blood transfusions. About 17.5% of patients were underweight. Around thirteen percent (12.5%) of adults were alcohol drinkers (Table 3).

**Table 3:** Socio-demographic characteristics of patients with acute lymphoblastic leukaemia.

Variables	Categorized variables	Number	Percent (%)
Sex	Males	22	55
	Females	18	45
Children (in years)	1–9	16	40
	10–14	4	10
Children (in year)	1–9	16	40
	10–17	8	20
Adults	≥ 18	16	40
Underweight	Yes	7	17.5
	No	33	82.5
Blood transfusion	Yes	35	87.5
	No	5	12.5
Alcohol intake	Yes	5	12.5
	No	35	87.5

### The incidence of chemotherapy drug-induced hepatotoxicity in patients with acute lymphoblastic leukemia

In each of the three measurements (at baseline, second and fourth weeks), a total of forty patients were involved for up to four weeks. Clinically significant hepatotoxicity, including moderate drug-induced liver injury, occurred in 15% of patients. Five percent of patients had mild drug-induced liver injuries. At the baseline and second-week measures, no patients were found to fulfill the criteria for drug-induced liver injury. There were no cases of severe or fatal drug-induced liver injury (Table 4).

**Table 4:** The grading of chemotherapeutic drug-induced liver injury in patients with acute lymphoblastic leukemia.

Grading	Severity	Number of patients	Percent
1	Mild	2	5
2	Moderate	6	15
3	Sever	–	–
4	Fatal	–	–

Dash (–) denotes absence.

### The effect of induction chemotherapeutic drugs on the alanine aminotransferase (ALT) enzyme in patients with acute lymphoblastic leukemia

**Figure 1:**
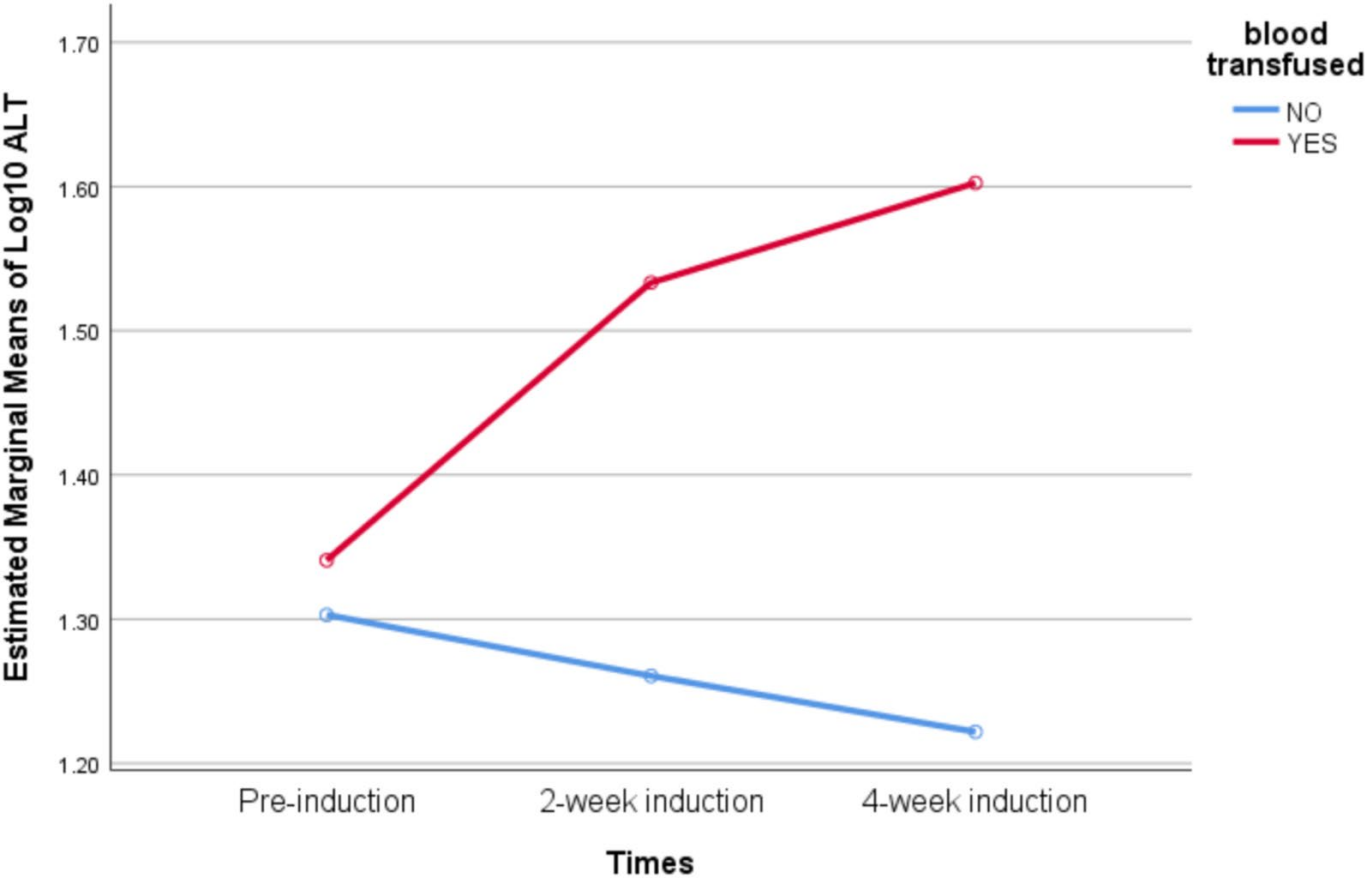
Depicts the interaction nature of ALT levels in patients with and without blood 465 transfusion during the induction period.

The assumption of sphericity was not met by Mauchly’s sphericity test (*p* = 0.002). A Greenhouse–Geisser correction was thus used (ɛ = 0.77). The ALT levels differed between those who received blood transfusions and those who did not receive blood transfusions during chemotherapeutic induction (F(1.6, 59) = 4.2, *p* = 0.029). The ALT level in patients who received blood transfusions increased significantly from pre-induction to 2-week induction and 4-week induction (*p* < 0.01), but not between 2-week induction and 4-week induction (*p* = 0.151). The elevation in ALT levels in those who did not get blood transfusions was not statistically significant (*p* = 0.99) (Table 5). Figure 1 depicts the interaction nature of chemotherapeutic-induced ALT levels in patients with and without blood transfusion.

**Table 5:** Compares the mean alanine aminotransferase before and after induction.

Blood transfused	Measurement time (Mean ± S.D)	Measurement time (Mean ± S.D)	*p* value
Yes	Pre-induction (22 IU/L ± 1.66 IU/L)	2-weeks after induction (34.1 IU/L ± 2.1 IU/L)	0.001
		4-weeks after induction (40.1 IU/L ± 2.0 IU/L)	0.001
	2-weeks after induction (34.1 IU/L ± 2.1 IU/L)	4-weeks after induction (40.1 IU/L ± 2.0 IU/L)	0.151
No	Pre-induction (20.1 IU/L ± 1.86 IU/L)	2-weeks after induction (18.2 IU/L ± 1.79 IU/L)	0.99
		4-weeks after induction (16.7 IU/L ± 1.4 IU/L)	0.99
	2-weeks after induction (18.2 IU/L ± 1.79 IU/L)	4-weeks after induction (16.7 IU/L ± 1.4 IU/L)	0.99

### The effect of induction chemotherapeutic drugs on aspartate aminotransferase (AST) enzyme in patients with acute lymphoblastic leukaemia

The assumption of sphericity was violated (*p* = 0.004). Therefore, the Greenhouse–Geisser adjustment was used (ɛ = 0.793). A statistically significant difference was observed between the three mean AST enzymes (F(1.6, 60.3) = 8.8, *p* = 0.001). The mean AST level significantly differed between pre-induction and 2 weeks of induction (*p* = 001), as well as between pre-induction and 4 weeks of induction (*p* = 0.014), but not statistically significant between 2-weeks and 4-weeks of induction (*p* = 0.99) (Table 6).

**Table 6:** Compares the mean aspartate aminotransferase before and after induction.

Measurement time (Mean ± S.D)	Measurement time (Mean ± S.D)	*p* value
Pre-induction (20.7 IU/L ± 1.7 IU/L)	2-weeks after induction (29.4 IU/L ± 1.9 IU/L)	0.001
	4-weeks after induction (29.2 IU/L ± 2.2 IU/L)	0.014
2-weeks after induction (29.4 IU/L ± 1.9 IU/L)	4-weeks after induction (29.2 IU/L ± 2.2 IU/L)	0.99

### The effect of induction chemotherapy on alkaline phosphate (ALP) enzyme in patients with acute lymphoblastic leukemia

The Mauchly’s sphericity test indicates that the condition of sphericity was not met (*p* = 0.001). Thus, the Greenhouse–Geisser correction was applied (ɛ = 0.687). There is a significant difference between the three mean ALP enzymes (F(1.4, 52.2) = 9.03, *p* = 0.002). A significant difference was seen between pre-induction ALP enzyme and 4-weeks of induction ALP enzyme (*p* = 0.006), and also between 2-week induction and 4-week induction (*p* = 0.029). However, no statistically significant difference was found between the pre-induction ALP enzyme and the 2-week induction ALP enzyme (*p* = 0.05). Ignoring all other factors, the mean ALP differs significantly between children and adults (F(1, 38) = 8.07, *p* = 0.007) (Table 7).

**Table 7:** Compares the mean of alkaline phosphate before and after induction.

Measurement time (Mean ± S.D)	Measurement time (Mean ± S.D)	*p* value
Pre-induction (219.1 IU/L ± 1.7 IU/L)	2-weeks after induction (252.7 IU/L ± 1.75 IU/L)	0.05
	4-weeks after induction (282.9 IU/L ± 1.9 IU/L)	0.006
2-weeks after induction (252.7 IU/L ± 1.75 IU/L)	4-weeks after induction (282.9 IU/L ± 1.9 IU/L)	0.029
Children 300 ± 1.64	Adults 191 ± 1.63	0.007

### The effect of chemotherapy induction on total bilirubin in patients with acute lymphoblastic leukemia

The variance homogeneity (*p* = 0.001) was violated. The Greenhouse–Geisser correction (ɛ = 0.72) was used. At least one significant difference exists between the three total bilirubin measures (baseline, 2-week induction, and 4-week induction) (F(1.4, 54.5) = 5.4, *p* = 0.014). Only total bilirubin differs significantly between pre-induction and 4 weeks of induction (*p* = 0.043). Controlling all other factors, total bilirubin in adults is significantly higher than in children (F(1, 38) = 7.1, *p* = 0.011) (Table 8).

**Table 8:** The comparison of the mean of total bilirubin before and after induction.

Measurement time (Mean ± S.D)	Measurement time (mean ± S.D)	*p* value
Pre-induction (0.6 IU/L ± 1.9 IU/L)	2-weeks after induction (0.76 IU/L ± 1.15 IU/L)	0.11
	4-weeks after induction (0.87 IU/L ± 2.4 IU/L)	0.043
2-weeks after induction (0.76 IU/L ± 1.15 IU/L)	4-weeks after induction (0.87 IU/L ± 2.4 IU/L)	0.312
Children 0.56 ± 1.86	Adults 0.96 ± 1.85	0.011

### The effect of chemotherapy induction on direct bilirubin in patients with acute lymphoblastic leukaemia

The assumption of sphericity was met (*p* = 0.81). A mixed model ANOVA revealed that there is at least one statistically significant difference between the three mean direct bilirubin levels (F(2, 76) = 19, *p* = 0.001). The Bonferroni post hoc test indicated that direct bilirubin levels differed significantly between pre-induction and post-induction (2 and 4 weeks of induction) (*p* < 0.01), but not between 2-weeks and 4-weeks of induction (*p* = 0.99) (Table 9).

**Table 9:** Compares the mean of direct bilirubin before and after induction.

Measurement time (Mean ± S.D)	Measurement time (Mean ± S.D)	*p* value
Pre-induction (0.25 mg/dl ± 2.3 mg/dl)	2-weeks after induction (0.47 mg/dl ± 2.3 mg/dl)	0.001
	4-weeks after induction (0.48 mg/dl ± 2.6 mg/dl)	0.001
2-weeks after induction (0.47 mg/dl ± 2.3 mg/dl)	4-weeks after induction (0.48 mg/dl ± 2.6 mg/dl)	0.99

## Discussion

This study was the first to evaluate the side effects of chemotherapeutic drugs on the liver during induction in Ethiopia. After chemotherapy induction, the mean of all liver function tests increased. The cause of the abnormal rise of these liver functions could be exposure to chemotherapy induction. This evidence supports the previous studies^9,12,16,17,25^. The incidence of chemotherapeutic drug-induced hepatotoxicity, which included moderate drug-induced liver injury, occurred in 15% of patients. These patients fully meet the criteria for clinically significant abnormalities in liver function tests^11–14^, indicating hepatocellular injury^10,12^. The mild drug-induced liver injury occurred in 5% of patients. The incidence of hepatotoxicity in this study is higher than in a prior studies in Egypt^21^ and Singapore^18^ but lower than in studies in the United States^19^ and the Netherlands^20^ for patients who received L-asparaginase as a chemotherapy induction. The difference could be attributed to the administration of four chemotherapy drugs in the current study, variations in hepatotoxicity grading criteria, the health status of patients, standard care of hospitals, and sample size. Furthermore, genetics can play a role in a significant difference in pancreatitis incidence^23^. ALT levels increased significantly in those who received blood transfusions from pre-induction to two weeks and four weeks of induction but not between two and four weeks of induction. For those without a blood transfusion, ALT levels have not increased significantly. Elevated levels of ALT in patients who have received blood transfusions may be caused by patients bleeding before donating blood, as these bleeding patients may have hepatotoxicity^23^. The levels of AST, direct bilirubin, and children's ALT differed significantly between pre-and post-induction but not between two and four weeks. This evidence suggests that chemotherapeutic drugs exposure triggers the production of AST, ALT and direct bilirubin, but increasing cumulative chemotherapy drug dosages may not increase the level of AST, ALT and direct bilirubin. This finding is also in line with a prior study that showed L-asparaginase dosages had no effect on liver toxicity^23^ and another study that found asparaginase activity-induced statistically significant increases in ALT and bilirubin^20^. ALP levels increased significantly from pre-induction to four weeks of induction, as well as from two weeks to four weeks of induction. Total bilirubin levels differed only significantly between pre-induction and four-week induction. This showed that increasing the cumulative chemotherapy drug dosages increased ALP and total bilirubin levels. These findings contradicted the previous finding^23^. Ignoring all other factors, children's ALP levels are significantly higher than those of adults, but the total bilirubin of adults is higher than that of children. The cause of the difference between ALP and total bilirubin may be biological; that is, for children, the concentration of ALP correlates with bone growth and the occurrence of a high concentration of total bilirubin in adults may be associated with increased age. According to the current studies, chemotherapy-induced hepatotoxicity was not significantly different between children and adults. This finding is against with the previous findings that reported L-asparaginase-induced hepatotoxicity differs between children and adults^24,25^. Genetic variation^23^ and sample size could be the contributing factors to these contradicting results. Although elevated serum liver enzymes and bilirubin showed liver injury, this hepatocellular injury may be transient due to the absence of additional liver parameters such as albumin and prothrombin, which indicated the overall liver function^12^.

Despite the fact that logistic regression was not used to control for confounding due to the small sample size, a mixed model ANOVA statistical test can aid in determining the relationship or interaction of independent variables with the dependent variable. Measuring the same group three times and comparing results can help to avoid variations between groups. This study helped clinicians understand chemotherapy drug induction-hepatotoxicity and the development of early preventive measures to limit the progression of drug-induced liver injury. However, patients with acute lymphoblastic leukemia are treated with combination chemotherapy, making it difficult to identify the specific drug causing the hepatic injury. The study is a baseline for future research to identify a precise drug agent causing liver injury with large sample size and a long follow-up period.

## Conclusion

A significant proportion of patients had clinically significant hepatotoxicity. The mean rise of liver function tests from baseline to post-induction is statistically significant. The ALT level increased significantly in those who received blood, but not in those who did not donate blood. ALP and total bilirubin levels differ between children and adults, regardless of other factors. The increase in liver enzymes during chemotherapy induction may be transient, resulting in no long-term liver injury. During induction, the chemotherapeutic drugs should be given without causing significant changes in liver function, and patients should be closely monitored.

## Supplementary Information

Supplementary Information.

## Data Availability

All materials and data were in the manuscript and supplementary material table (Table S1).

## Abbreviations

ANOVA: Analysis of Variance
SPSS: Statically Package for Social Science
S.D: Standard deviation
AST: Aspartate amino transferase
ALT: Alanine amino transferase
ALP: Alkaline phosphate
ULN: Upper limit normal
