# Supplementary material for: Evaluation of liver function tests to identify hepatotoxicity among acute lymphoblastic leukemia patients who are receiving chemotherapy induction
Source: Sci Rep. 2022 Aug 2;12:13215. doi: 10.1038/s41598-022-17618-w (PMC9346124; doi:10.1038/s41598-022-17618-w)
Supplement: Supplementary file 1 — Supplementary Information. [file 41598_2022_17618_MOESM1_ESM.docx]

| Supplementary material Table-Table S1: Shows the Laboratory values of liver function tests. | | | | | | | | | | | | | | | | |  |  |  |  |  |  |  |  |  |  |  |  |
| --- | --- | --- | --- | --- | --- | --- | --- | --- | --- | --- | --- | --- | --- | --- | --- | --- | --- | --- | --- | --- | --- | --- | --- | --- | --- | --- | --- | --- |
| **ID** | **Adult & pediatric** | **sex** | **age** | **blood transfused** | **Under**  **weight** | **Alcohol**  **intake** | AST1 | AST2 | AST3 | ALT1 | ALT2 | ALT3 | ALP1 | ALP2 | ALP3 |  |  |  |  |  |  |  |  |  |  |  |  |  |
| 1 | adult | female | 18 | YES | yes | no | 34 | 54 | 36 | 38 | 62 | 61 | 256 | 311 | 323 |  |  |  |  |  |  |  |  |  |  |  |  |  |
| 2 | adult | female | 17 | YES | No | no | 17 | 14 | 10 | 33 | 39 | 14 | 198 | 142 | 97 |  |  |  |  |  |  |  |  |  |  |  |  |  |
| 3 | adult | male | 15 | YES | No | no | 38 | 64 | 67 | 23 | 65 | 91 | 96 | 124 | 176 |  |  |  |  |  |  |  |  |  |  |  |  |  |
| 4 | adult | male | 24 | YES | No | no | 36 | 60 | 52 | 40 | 48 | 58 | 144 | 225 | 308 |  |  |  |  |  |  |  |  |  |  |  |  |  |
| 5 | adult | male | 21 | YES | yes | no | 17 | 12 | 14 | 26 | 23 | 35 | 105 | 120 | 133 |  |  |  |  |  |  |  |  |  |  |  |  |  |
| 6 | adult | male | 17 | YES | No | no | 14 | 6 | 12 | 7 | 11 | 15 | 116 | 187 | 220 |  |  |  |  |  |  |  |  |  |  |  |  |  |
| 7 | adult | female | 29 | YES | No | no | 11 | 19 | 45 | 21 | 35 | 57 | 124 | 201 | 302 |  |  |  |  |  |  |  |  |  |  |  |  |  |
| 8 | adult | male | 20 | YES | No | no | 29 | 31 | 118 | 14 | 47 | 167 | 313 | 204 | 253 |  |  |  |  |  |  |  |  |  |  |  |  |  |
| 9 | adult | female | 50 | NO | yes | no | 9 | 16 | 16 | 10 | 24 | 29 | 98 | 166 | 143 |  |  |  |  |  |  |  |  |  |  |  |  |  |
| 10 | adult | female | 26 | YES | No | no | 34 | 27 | 23 | 40 | 39 | 34 | 153 | 101 | 167 |  |  |  |  |  |  |  |  |  |  |  |  |  |
| 11 | adult | male | 27 | YES | No | no | 14 | 18 | 11 | 14 | 34 | 21 | 134 | 271 | 267 |  |  |  |  |  |  |  |  |  |  |  |  |  |
| 12 | adult | male | 22 | NO | No | no | 40 | 87 | 89 | 40 | 35 | 11 | 176 | 171 | 128 |  |  |  |  |  |  |  |  |  |  |  |  |  |
| 13 | adult | female | 41 | YES | No | yes | 14 | 45 | 67 | 23 | 54 | 71 | 207 | 367 | 379 |  |  |  |  |  |  |  |  |  |  |  |  |  |
| 14 | adult | male | 28 | YES | No | yes | 20 | 29 | 43 | 37 | 81 | 89 | 131 | 178 | 209 |  |  |  |  |  |  |  |  |  |  |  |  |  |
| 15 | adult | male | 30 | YES | yes | yes | 17 | 44 | 39 | 32 | 41 | 44 | 209 | 254 | 699 |  |  |  |  |  |  |  |  |  |  |  |  |  |
| 16 | adult | female | 18 | YES | No | no | 5 | 17 | 8 | 15 | 11 | 27 | 105 | 74 | 104 |  |  |  |  |  |  |  |  |  |  |  |  |  |
| 17 | adult | female | 15 | YES | No | no | 10 | 23 | 31 | 14 | 32 | 49 | 393 | 427 | 466 |  |  |  |  |  |  |  |  |  |  |  |  |  |
| 18 | adult | male | 52 | NO | yes | yes | 22 | 13 | 9 | 37 | 23 | 18 | 155 | 142 | 96 |  |  |  |  |  |  |  |  |  |  |  |  |  |
| 19 | adult | male | 28 | YES | No | no | 29 | 43 | 55 | 36 | 72 | 101 | 278 | 211 | 389 |  |  |  |  |  |  |  |  |  |  |  |  |  |
| 20 | adult | male | 28 | YES | No | yes | 12 | 11 | 18 | 35 | 38 | 60 | 103 | 307 | 394 |  |  |  |  |  |  |  |  |  |  |  |  |  |
| 21 | pediatric | female | 9 | YES | No | No | 16 | 34 | 41 | 10 | 12 | 19 | 354 | 364 | 369 |  |  |  |  |  |  |  |  |  |  |  |  |  |
| 22 | pediatric | female | 12 | YES | No | No | 19 | 31 | 14 | 29 | 18 | 21 | 111 | 129 | 130 |  |  |  |  |  |  |  |  |  |  |  |  |  |
| 23 | pediatric | female | 6 | NO | No | No | 9 | 16 | 11 | 17 | 13 | 14 | 340 | 780 | 928 |  |  |  |  |  |  |  |  |  |  |  |  |  |
| 24 | pediatric | male | 5 | YES | No | No | 36 | 45 | 67 | 15 | 39 | 71 | 334 | 386 | 412 |  |  |  |  |  |  |  |  |  |  |  |  |  |
| 25 | pediatric | male | 2 | YES | No | No | 32 | 29 | 8 | 13 | 14 | 9 | 326 | 250 | 182 |  |  |  |  |  |  |  |  |  |  |  |  |  |
| 26 | pediatric | male | 5 | YES | No | No | 11 | 36 | 48 | 8 | 22 | 32 | 393 | 769 | 866 |  |  |  |  |  |  |  |  |  |  |  |  |  |
| 27 | pediatric | male | 10 | YES | yes | No | 39 | 22 | 18 | 24 | 19 | 16 | 633 | 462 | 445 |  |  |  |  |  |  |  |  |  |  |  |  |  |
| 28 | pediatric | female | 10 | YES | No | No | 56 | 76 | 79 | 27 | 37 | 61 | 99 | 132 | 169 |  |  |  |  |  |  |  |  |  |  |  |  |  |
| 29 | pediatric | female | 5 | YES | No | No | 15 | 22 | 40 | 18 | 28 | 21 | 187 | 241 | 371 |  |  |  |  |  |  |  |  |  |  |  |  |  |
| 30 | pediatric | male | 2 | YES | No | No | 36 | 67 | 39 | 37 | 100 | 69 | 337 | 294 | 278 |  |  |  |  |  |  |  |  |  |  |  |  |  |
| 31 | pediatric | male | 3 | YES | No | No | 20 | 74 | 81 | 12 | 44 | 55 | 283 | 319 | 323 |  |  |  |  |  |  |  |  |  |  |  |  |  |
| 32 | pediatric | male | 7 | YES | No | No | 34 | 29 | 26 | 40 | 88 | 36 | 295 | 364 | 312 |  |  |  |  |  |  |  |  |  |  |  |  |  |
| 33 | pediatric | female | 6 | YES | No | No | 33 | 37 | 11 | 25 | 12 | 19 | 209 | 228 | 243 |  |  |  |  |  |  |  |  |  |  |  |  |  |
| 34 | pediatric | female | 9 | YES | No | No | 18 | 55 | 59 | 13 | 47 | 67 | 590 | 611 | 676 |  |  |  |  |  |  |  |  |  |  |  |  |  |
| 35 | pediatric | male | 13 | YES | No | No | 7 | 9 | 15 | 16 | 19 | 13 | 245 | 223 | 215 |  |  |  |  |  |  |  |  |  |  |  |  |  |
| 36 | pediatric | female | 5 | NO | No | No | 32 | 17 | 16 | 13 | 8 | 16 | 345 | 171 | 102 |  |  |  |  |  |  |  |  |  |  |  |  |  |
| 37 | pediatric | female | 6 | YES | No | No | 22 | 31 | 14 | 39 | 48 | 84 | 482 | 563 | 959 |  |  |  |  |  |  |  |  |  |  |  |  |  |
| 38 | pediatric | female | 3 | YES | yes | No | 39 | 53 | 71 | 36 | 71 | 82 | 219 | 261 | 301 |  |  |  |  |  |  |  |  |  |  |  |  |  |
| 39 | pediatric | male | 3 | YES | No | No | 33 | 49 | 61 | 31 | 39 | 57 | 420 | 623 | 839 |  |  |  |  |  |  |  |  |  |  |  |  |  |
| 40 | pediatric | male | 6 | YES | No | No | 28 | 43 | 59 | 16 | 23 | 23 | 397 | 453 | 484 |  |  |  |  |  |  |  |  |  |  |  |  |  |

| **BLI**BLIT1**T1** | BLIT2 | BLIT3 | BLID1 | BLID2 | BLID3 |
| --- | --- | --- | --- | --- | --- |
| 1.66 | 1.6800 | 1.7900 | 1.5500 | 1.5900 | 1.6900 |
| 1.56 | 0.3900 | 0.3200 | 0.3100 | 0.1900 | 0.3100 |
| 0.2 | 0.3300 | 0.4900 | 0.0900 | 0.1900 | 0.1810 |
| 0.34 | 0.4900 | 0.9000 | 0.0700 | 0.2900 | 0.1200 |
| 1.24 | 0.2100 | 0.1600 | 0.2700 | 0.2000 | 0.1100 |
| 0.17 | 0.2100 | 0.3000 | 0.0400 | 0.1400 | 0.2900 |
| 0.81 | 1.9100 | 2.4200 | 0.1600 | 1.1300 | 2.0100 |
| 0.76 | 0.6900 | 0.9300 | 0.1600 | 0.2800 | 0.3300 |
| 1.29 | 2.7400 | 3.4500 | 1.1700 | 1.5700 | 2.8700 |
| 0.84 | 0.4100 | 0.3000 | 0.2200 | 0.1000 | 0.2700 |
| 0.7 | 0.9400 | 2.0900 | 0.3600 | 1.9000 | 1.9800 |
| 1.25 | 1.4500 | 1.9300 | 0.2500 | 1.4300 | 0.2200 |
| 1.15 | 2.1000 | 2.0500 | 2.7500 | 1.7700 | 2.6800 |
| 0.96 | 1.3400 | 1.3700 | 0.2000 | 0.9000 | 1.3500 |
| 0.75 | 0.7500 | 1.1300 | 0.2100 | 0.3600 | 0.6500 |
| 0.46 | 0.5700 | 0.3100 | 0.0800 | 0.4000 | 0.1200 |
| 0.75 | 0.9100 | 1.4000 | 0.6100 | 0.7400 | 0.8800 |
| 1.12 | 0.4200 | 0.1500 | 0.1600 | 0.3000 | 0.0900 |
| 1.13 | 1.7000 | 2.2200 | 0.4100 | 1.6000 | 1.9000 |
| 1.07 | 1.1000 | 1.7800 | 0.2300 | 1.0600 | 0.5400 |
| 0.38 | 1.2000 | 1.2900 | 0.2400 | 0.3000 | 0.4100 |
| 0.24 | 0.8900 | 0.3900 | 0.1300 | 0.2500 | 0.1700 |
| 0.27 | 0.4100 | 0.4600 | 0.1100 | 0.1600 | 0.3100 |
| 0.23 | 0.5900 | 1.6700 | 0.2100 | 0.3100 | 0.9900 |
| 0.71 | 1.8000 | 0.5000 | 0.2000 | 1.2700 | 0.2300 |
| 0.32 | 0.6100 | 1.1000 | 0.2900 | 0.3300 | 0.4100 |
| 0.6 | 0.5000 | 0.5300 | 0.3700 | 0.3000 | 0.3400 |
| 0.81 | 0.9900 | 1.2100 | 0.3900 | 0.7700 | 0.7100 |
| 0.18 | 0.2800 | 0.3200 | 0.1200 | 0.2100 | 0.2000 |
| 3 | 2.4900 | 2.4700 | 0.9400 | 0.8700 | 0.7100 |
| 0.45 | 0.8200 | 1.4600 | 0.3100 | 0.4200 | 0.7300 |
| 0.24 | 0.3300 | 0.5200 | 0.1800 | 0.2100 | 0.2500 |
| 0.28 | 0.2200 | 0.1500 | 0.2200 | 0.0900 | 0.1400 |
| 0.38 | 0.4900 | 1.3400 | 0.3300 | 0.2000 | 0.9900 |
| 0.19 | 0.2800 | 0.8500 | 0.1000 | 0.2400 | 0.4200 |
| 0.29 | 0.4100 | 0.6100 | 0.1700 | 0.2200 | 0.3200 |
| 0.28 | 0.2900 | 0.3400 | 0.1700 | 0.1900 | 0.1600 |
| 0.25 | 1.2400 | 1.4200 | 0.2000 | 0.8800 | 1.1400 |
| 1.28 | 1.4100 | 1.4300 | 0.6400 | 0.7800 | 1.0900 |
| 0.34 | 1.3800 | 1.5300 | 0.2600 | 0.7700 | 0.7900 |

| Total sample size =40 |
| --- |
| 1= pretreatment measurement |
| 2=2nd weeks measurement |
| 3 =4th weeks measurement |
| BLI-T=total bilirubin |
| BLI-D= direct bilirubin |
| AST=aspartate aminotransferase |
| ALT=alanine aminotransferase |
| ALP=alkaline phosphate |
